# SGLT2 Inhibition as a Perioperative Cardiorenal Stabilizer in Cardiac Surgery: Integrated Clinical Cohort and Pleiotropic Network-Based Pharmacological Analysis

**DOI:** 10.3390/jcm15082873

**Published:** 2026-04-10

**Authors:** Lutfi Cagatay Onar, Ersin Guner, Ibrahim Yilmaz

**Affiliations:** 1 Department of Cardiovascular Surgery, Republic of Turkey, Ministry of Health, Dr. Ismail Fehmi Cumalioglu City Hospital, Tekirdag 59020, Turkey; lutficagatay.onar@saglik.gov.tr; 2 Department of Pharmacy, Republic of Turkey, Ministry of Health, Konya Numune Hospital, Konya 42060, Turkey; drersinguner@gmail.com; 3 Unit of Pharmacovigilance, Republic of Turkey, Ministry of Health, Dr. Ismail Fehmi Cumalioglu City Hospital, Tekirdag 59020, Turkey

**Keywords:** cardiac surgery, SGLT2 inhibitors, rehospitalization, inverse probability weighting, molecular docking, protein–protein interaction, hub proteins, functional enrichment

## Abstract

**Background**: Patients with type 2 diabetes mellitus (T2DM) undergoing cardiac surgery represent a high-risk population characterized by substantial cardiometabolic stress and increased susceptibility to postoperative heart failure, renal dysfunction, and unplanned rehospitalization. Although sodium-glucose cotransporter 2 (SGLT2) inhibitors provide established cardiorenal protection in ambulatory populations, their perioperative impact in cardiac surgery cohorts remains insufficiently defined. **Methods**: In a single-center retrospective cohort of 620 T2DM patients, inverse probability of treatment weighting and time-dependent Cox regression were applied to account for perioperative treatment interruption and delayed postoperative reinitiation when evaluating the association between chronic SGLT2 inhibitor therapy and 12-month rehospitalization risk. To provide biological context for the observed clinical associations, target-driven systems pharmacology, molecular docking against SGLT2, NHE1, AMPK, and NLRP3, and protein–protein interaction (PPI) network analysis were performed. Hub proteins were identified using Maximal Clique Centrality, followed by functional enrichment (GO/KEGG) analysis. **Results**: Chronic SGLT2 inhibitor therapy was associated with reduced first rehospitalization (HR 0.64; 95% CI 0.48–0.85; *p* = 0.002) and a lower cumulative rehospitalization burden (IRR 0.61; 95% CI 0.46–0.82; *p* = 0.001), primarily driven by heart failure-related and metabolic phenotypes. Molecular docking analyses identified favorable binding with SGLT2 and additional cardiometabolic and inflammatory targets, including NHE1, AMPK, NLRP3, IKKβ, IL-6Rα, and PPAR isoforms, suggesting modulation of myocardial ion homeostasis, metabolic resilience, and inflammatory signaling. PPI analysis identified eight hub proteins (AKT1, MTOR, STAT3, EGFR, PIK3CA, SRC, MAPK1, and MAPK3) significantly enriched in PI3K/AKT, MAPK/ERK, and ErbB signaling pathways. **Conclusions**: Chronic SGLT2 inhibitor therapy was independently associated with reduced postoperative rehospitalization and cumulative event burden in T2DM patients undergoing cardiac surgery. Integrated in silico analyses offer mechanistic hypotheses consistent with the observed clinical associations. These findings suggest that structured perioperative SGLT2 inhibitor management may contribute to improved postoperative outcomes, while prospective validation in future studies would strengthen these findings. However, given the retrospective observational design, these findings should be interpreted as associative rather than causal.

## 1. Introduction

Type 2 diabetes mellitus (T2DM) remains a major independent risk factor for adverse outcomes following cardiac surgery, characterized by complex cellular interplay between cardiomyocytes and non-myocytes [1]. Despite advances in surgical techniques, diabetic patients face an increased risk of cardiac surgery-associated acute kidney injury and subsequent renal dysfunction [2,3]. Renal dysfunction is highly prevalent in patients with diabetes and cardiovascular disease and represents a major determinant of prognosis, particularly in those with heart failure. Diabetes contributes substantially to chronic kidney disease progression, which is characterized by persistent reductions in estimated glomerular filtration rate and increased urinary protein excretion, thereby increasing perioperative cardiorenal vulnerability in cardiac surgery populations [4]. Beyond these acute complications, managing the long-term clinical trajectory of these patients requires adherence to updated heart failure guidelines, which now emphasize the necessity of specialized pharmacological interventions to reduce the risk of clinical deterioration [5]. In this context, understanding the vascular and pleiotropic effects of newer cardiometabolic therapies such as Sodium-glucose cotransporter 2 (SGLT2) inhibitors is crucial for improving postsurgical outcomes and mitigating long-term cardiorenal failure [6]. SGLT2 inhibitors have consistently demonstrated significant reductions in heart failure-related hospitalization and clinically meaningful renal endpoints across large cardiovascular outcome trials and dedicated heart failure studies [5,7,8,9].

These benefits extend beyond glycemic control and include osmotic diuresis, natriuresis, improved ventricular loading conditions, and the modulation of myocardial energetics [6,8]. Emerging evidence from systematic reviews and meta-analyses suggests that SGLT2 inhibition may also indirectly modulate kinase-mediated signaling and protein interaction networks, underlying cardiovascular and renal protection independently of glucose lowering [7,8,9]. These mechanistic effects are particularly relevant during the early and intermediate postoperative period following cardiac surgery, a phase characterized by neurohormonal activation, fluid shifts, and systemic inflammation [2].

Despite these theoretical advantages, evidence on the impact of chronic SGLT2 inhibitor therapy in cardiac surgery patients remains limited. However, data specifically addressing the perioperative management of chronic SGLT2 inhibitor therapy in cardiac surgery populations remain limited, particularly regarding the impact of temporary perioperative interruption and subsequent reinitiation on postoperative morbidity and rehospitalization risk. Landmark SGLT2 trials generally excluded perioperative populations, leaving a gap in understanding how pre-existing therapy may influence postoperative rehospitalization and cumulative event burden [5,6,7,8,9,10,11].

To provide additional biological context for the observed clinical associations, exploratory in silico approaches were incorporated as hypothesis-generating analyses rather than confirmatory mechanistic investigations. Molecular docking, protein–protein interaction (PPI) network analysis, and functional enrichment were used to identify biologically plausible pathways potentially associated with the observed clinical outcomes. These analyses were designed to be hypothesis-generating and supportive, rather than confirmatory, acknowledging that in silico findings do not establish causal relationships but may offer mechanistic plausibility for clinical associations. Observational treatment-effect analyses are susceptible to confounding and immortal time bias [11]. Therefore, bias-aware approaches, including IPTW, time-dependent exposure modeling, and competing risk regression, were applied, and findings were interpreted as associative rather than causal. Against this background, we conducted an integrated clinical and exploratory in silico analysis to evaluate potential associations and provide a mechanistic context.

Given the pleiotropic cardiometabolic effects of SGLT2 inhibition, exploring biologically plausible signaling pathways may help contextualize observed clinical associations. Although SGLT2 is primarily expressed in the renal proximal tubule, accumulating evidence indicates indirect myocardial and vascular effects mediated through hemodynamic modulation, shifts in metabolic substrate utilization, and attenuation of inflammatory signaling [6,7]. These mechanisms may contribute to observed reductions in heart failure hospitalization and renal endpoints in both diabetic and non-diabetic populations [7,8,9]. In the present study, we employed PPI network mapping, Gene Ontology (GO) and pathway enrichment, and Maximal Clique Centrality (MCC) analysis to highlight key molecular nodes, complemented by molecular docking to explore potential interactions of SGLT2 inhibitors with cardiometabolic and inflammatory targets. While these analyses provide biologically plausible hypotheses, they do not confirm direct intracellular pharmacologic engagement, as systemic drug exposure, tissue distribution, and target accessibility differ substantially from in silico conditions.

The primary aim of this study was to evaluate the association between chronic SGLT2 inhibitor therapy and 12-month postoperative rehospitalization in patients with T2DM undergoing cardiac surgery. An integrated clinical and exploratory in silico approach was used to provide a mechanistic context, and findings were interpreted as associative rather than causal.

## 2. Materials and Methods

### 2.1. Study Design and Patient Population

This study was designed as a single-center retrospective cohort analysis conducted at a tertiary referral cardiovascular surgery center. Adult patients with a documented diagnosis of T2DM who underwent open heart surgery with cardiopulmonary bypass between the predefined study dates were eligible. Surgical procedures included coronary artery bypass grafting, valve surgery, or combined procedures performed via median sternotomy. To account for procedural heterogeneity, surgical characteristics, including procedure type (isolated CABG, valve surgery, or combined procedures), were incorporated into adjusted analyses and evaluated in prespecified sensitivity analyses.

Exclusion criteria were type 1 diabetes mellitus, end-stage renal disease requiring chronic dialysis, incomplete perioperative clinical data, or insufficient follow-up information regarding rehospitalization outcomes. The study protocol was approved by the local institutional ethics committee, and the requirement for informed consent was waived due to the retrospective observational design.

**Figure 1:**
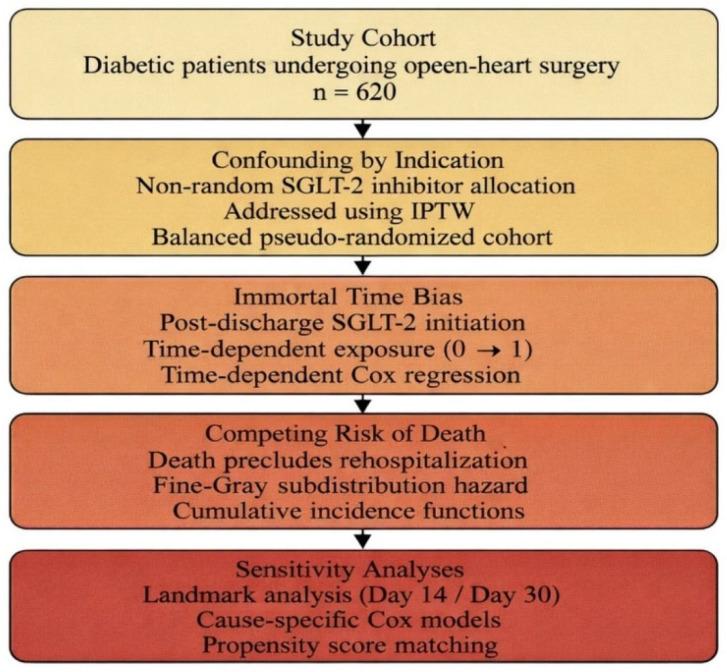
Study flow diagram and analytical framework.

To evaluate the association between chronic SGLT2 inhibitor therapy and postoperative rehospitalization while addressing major sources of bias relevant to observational surgical cohorts, the analytical framework incorporated bias-aware methods. Propensity scores were estimated using a multivariable logistic regression model in which chronic SGLT2 inhibitor use served as the dependent variable and baseline demographic, clinical, and surgical characteristics were included as covariates; inverse probability of treatment weighting based on these propensity scores was then applied to evaluate the association between chronic SGLT2 inhibitor therapy and postoperative rehospitalization while minimizing confounding. Immortal time bias was addressed using time-dependent exposure modeling, and death was treated as a competing event using Fine–Gray subdistribution hazard models. Sensitivity analyses, including landmark analyses and alternative cause-specific competing-risk models, were performed to assess the robustness of the findings. Stabilized weights were examined for extreme values, and the balance between groups after weighting was assessed using standardized mean differences. Missing data were minimal (<5% for all variables) and were handled using complete-case analysis. The patient selection, exposure classification, follow-up, and analytical workflow are summarized in Figure 1.

### 2.2. Treatment Exposure and Patient Stratification

Chronic SGLT2 inhibitor exposure was verified through institutional electronic prescription records and confirmed as continuous use for at least 90 days prior to surgery. Medication continuity was cross-checked using pharmacy dispensing data and perioperative medication charts to minimize exposure misclassification. Post-discharge continuation or reinitiation of therapy was documented separately according to routine clinical practice.

Chronic SGLT2 inhibitor therapy was defined as documented active use prior to surgery. In accordance with institutional perioperative practice, SGLT2 inhibitors were routinely discontinued approximately 48 h prior to cardiac surgery. Postoperatively, therapy was generally reinitiated at or shortly after hospital discharge, with a mean time to reinitiation of 7 ± 1.6 days, provided that patients had achieved adequate hemodynamic and metabolic stability. Given the retrospective design, exact timing varied across individual cases; therefore, treatment exposure was modeled as a time-dependent variable to account for perioperative discontinuation and delayed post-discharge reinitiation.

Patients not receiving SGLT2 inhibitors were managed with alternative glucose-lowering strategies, including insulin and/or other oral antidiabetic agents. Treatment allocation was determined solely by the treating physicians and was independent of study participation.

### 2.3. Clinical Outcomes, Definitions, and Follow-Up

The primary outcome of this study was the time to the first unplanned rehospitalization within 12 months following index cardiac surgery discharge. A secondary outcome was the cumulative burden of all-cause rehospitalizations during the same follow-up period, accounting for recurrent events. Rehospitalization was defined as any unplanned hospital admission lasting ≥24 h after discharge from the index cardiac surgery hospitalization. To ensure clinical granularity, rehospitalization events were further categorized into specific phenotypes:

Heart failure-related: Admissions for acute decompensated heart failure, requiring intravenous diuretic therapy or inotropic support.

Renal-related: Admissions for acute and acute-on-chronic kidney injury or electrolyte imbalances necessitating nephrological intervention.

Metabolic-related: Admissions for dysglycemia (severe hypoglycemia or diabetic ketoacidosis) or other metabolic disturbances.

Follow-up data were collected through a standardized review of the national health insurance database and institutional electronic records. For patients with multiple admissions, each event was recorded with its respective date and primary diagnosis to enable recurrent event modeling. Patients who died during the follow-up period without a prior rehospitalization were treated as a competing risk in the cumulative incidence analysis.

The primary outcome was defined as all-cause unplanned rehospitalization within 12 months after discharge from the index cardiac surgery hospitalization. These included, but were not limited to, worsening heart failure or congestion, arrhythmias requiring medical management, renal dysfunction or fluid imbalance, respiratory complications, electrolyte disturbances, wound or sternal complications, and other postoperative conditions considered clinically attributable to the index cardiac surgical procedure. Outcome events were identified through a review of institutional electronic medical records and national health system data, and rehospitalization diagnoses were classified according to the principal clinical reason for admission.

Secondary outcomes included recurrent rehospitalization events during follow-up, allowing assessment of the total rehospitalization burden rather than time to first event alone. All-cause mortality was treated as a competing event in analyses of rehospitalization to account for the potential influence of early postoperative death on cumulative incidence estimates. Whenever available, causes of rehospitalization were classified as heart failure related, cardiorenal, arrhythmic, or other cardiovascular causes in order to facilitate clinical interpretation of the principal drivers of rehospitalization. Recurrent rehospitalization events were analyzed using negative binomial count-based regression models, and incidence rate ratios (IRRs) were calculated to quantify cumulative event burden.

### 2.4. Statistical Analysis

All statistical analyses were conducted according to a prespecified analytical plan using IBM SPSS Statistics (Version 30; IBM Corp., Armonk, NY, USA). The analytical strategy was specifically designed to evaluate the association between chronic SGLT2 inhibitor therapy and postoperative rehospitalization, while minimizing bias inherent to observational clinical data. Continuous variables were assessed for normality using visual inspection and statistical tests, and are presented as mean ± standard deviation or median with interquartile range, as appropriate. Categorical variables are summarized as counts and percentages. Baseline comparisons between treatment groups were performed using Student’s *t*-test or Mann–Whitney U test for continuous variables, and χ^2^ test or Fisher’s exact test for categorical variables, depending on data distribution and cell counts. Time-to-event outcomes were analyzed using survival analysis methods, with effect estimates reported as hazard ratios (HRs) and 95% confidence intervals (CIs). Rehospitalization events were identified through the institutional electronic health record system and verified by review of hospital admission records. To account for delayed initiation or continuation of SGLT2 inhibitor therapy, exposure was modeled as a time-dependent covariate. Competing-risk analyses were performed to address all-cause mortality as a competing event, and subdistribution hazard ratios are reported when applicable. For recurrent rehospitalization events, negative binomial count-based regression models were employed, IRRs were calculated, and model assumptions were assessed to ensure validity of the estimates.

#### 2.4.1. Baseline Comparisons

Baseline demographic, clinical, laboratory, and surgical characteristics were summarized according to treatment group. Continuous variables are presented as mean ± standard deviation or median with interquartile range, as appropriate, and categorical variables as counts and percentages.

Given the broad clinical effects of SGLT2 inhibitors, which are increasingly recognized to arise from pleiotropic and incompletely characterized mechanisms, it was essential to adopt a methodological framework that minimizes bias beyond conventional observational designs. Specifically, confounding by indication, immortal time bias due to delayed treatment initiation, and competing mortality posed a risk of spurious associations if not properly addressed.

Accordingly, the analytical strategy incorporated time-dependent exposure definitions, competing-risk modeling, and multiple sensitivity analyses. This approach was intended to ensure that observed associations reflect genuine differences in postoperative morbidity rather than artifacts arising from treatment selection, survival bias, or differential follow-up.

Given the non-randomized nature of treatment allocation, patients were screened according to predefined inclusion and exclusion criteria. To address confounding by indication, IPTW based on propensity scores was applied. IPTW generated a weighted pseudo-population that balanced baseline characteristics across treatment groups, thereby reducing bias in effect estimates. The propensity score model included age, sex, body mass index (BMI), HbA1c, insulin use, chronic kidney disease, hypertension, left ventricular ejection fraction, and surgical procedure type.

Covariates were selected based on established determinants of postoperative cardiorenal risk and variables associated with treatment allocation in observational SGLT2 inhibitor studies. Stabilized weights were applied to improve precision, and covariate balance between treatment groups was assessed using standardized mean differences, with values < 0.1 considered indicative of adequate balance.

Overlap of propensity score distributions between treatment groups was visually assessed, and no evidence of substantial non-overlap or extreme weight instability was observed.

#### 2.4.2. Time-Dependent Exposure and Immortal Time Bias

To mitigate immortal time bias arising from post-discharge initiation or continuation of SGLT2 inhibitor therapy, treatment exposure was modeled as a time-dependent covariate. Time-dependent Cox proportional hazards regression models were constructed in both unweighted and inverse probability of treatment weighting (IPTW)-weighted cohorts. Interaction terms were tested within IPTW-weighted Cox models as exploratory assessments to evaluate potential effect modification across clinically relevant subgroups. Hazard ratios (HRs) with 95% confidence intervals (CIs) were estimated to assess the association between SGLT2 inhibitor use and the risk of rehospitalization, accounting for the dynamic timing of treatment initiation.

#### 2.4.3. Competing Risk Modeling

Given the potential for postoperative mortality to preclude rehospitalization, competing risk analyses were performed using the Fine-Gray subdistribution hazard model. Death was treated as a competing event, allowing estimation of the cumulative incidence of rehospitalization while accounting for differences in mortality risk between treatment groups.

#### 2.4.4. Recurrent Event Analysis

To evaluate the total rehospitalization burden during follow-up, recurrent event analyses were performed.

Negative binomial regression models with log-transformed follow-up time included as an offset were used to estimate IRRs for rehospitalization events. Robust variance estimators were applied to account for within-patient correlation and repeated events.

#### 2.4.5. Sensitivity Analyses

Prespecified sensitivity analyses were conducted to assess the robustness of the findings across alternative modeling strategies and patient subgroups. These analyses included unweighted models, models restricted to specific surgical subtypes, and stratified analyses based on baseline cardiac and renal function.

### 2.5. In Silico Analyses

Molecular docking analyses were performed using AutoDock 4.2.6 software. Preparation of receptor proteins and ligands, definition of grid parameters, and execution of docking simulations were conducted in the AutoDockTools (MGLTools, version 1.5.6) environment.

For each protein–ligand pair, at least 10 independent docking runs were generated using the Lamarckian Genetic Algorithm. Resulting conformations were evaluated based on binding energies (ΔG_binding, kcal/mol), hydrogen bond interactions, and root-mean-square deviation (RMSD) values. Poses with the lowest binding energy and RMSD ≤ 2 Å were considered the most stable and reliable binding conformations. Detailed analysis of protein–ligand interactions, including hydrogen bond identification, was performed within the MGLTools environment.

Three-dimensional (3D) structures of the best docking poses were visualized using Python Molecular Viewer (PMV, version 1.5.6) to allow qualitative assessment of molecular interactions.

#### 2.5.1. Molecular Docking Analysis

To complement clinical findings and explore mechanistic plausibility, in silico molecular docking analyses were performed. Empagliflozin and dapagliflozin were selected as representative SGLT2 inhibitors based on their widespread clinical use.

In molecular docking analyses, these SGLT2 inhibitors were hypothesized to exert potential pleiotropic effects not only by suppressing renal glucose reabsorption but also by modulating energy metabolism, ion homeostasis, and inflammatory signaling pathways. Three-dimensional (3D) crystal structures of the target proteins were obtained from the Protein Data Bank (PDB; https://www.rcsb.org, accessed on 7 April 2026)) database.

As the primary target, ligand-bound human crystal SGLT2 structures were utilized, including PDB: 7VSI [12] for empagliflozin and PDB: 8HEZ [13] for dapagliflozin, to validate the specific binding modes of the drugs at the active site as well as their hydrogen bonding interactions. The docking protocol was validated by redocking the co-crystallized ligands into their respective binding sites, yielding RMSD values ≤ 2 Å, consistent with the predefined stability criteria.

In addition to SGLT2 (primary target), the molecular interactions of empagliflozin and dapagliflozin were evaluated with AMP-activated protein kinase (AMPK; PDB ID: 6B2E) [14], sodium-hydrogen exchanger 1 (NHE1; PDB ID: 7DSX) [15], IκB kinase β (IKKβ; PDB ID: 4KIK) [16], NOD-, LRR-, and pyrin domain-containing protein 3 (NLRP3; PDB ID: 8RI2) [17], interleukin-6 alpha receptor (IL-6Rα; PDB ID: 1P9M) [18], tumor necrosis factor receptor 1 (TNFR1; PDB ID: 7KPB) [19], peroxisome proliferator-activated receptor alpha (PPAR-α; PDB ID: 7BQ3) [20], and peroxisome proliferator-activated receptor gamma (PPAR-γ; PDB ID: 6MD4) [21], to investigate potential molecular-level interactions. These additional targets were selected to explore pathway-level biological plausibility rather than to imply direct pharmacological specificity of SGLT2 inhibitors toward each protein.

To ensure methodological transparency and reproducibility, the complete docking workflow, including receptor–ligand preparation, docking parameter settings, and target-specific binding site definitions for SGLT2, AMPK, IKKβ, IL-6Rα, NHE1, NLRP3, PPAR-α, PPAR-γ, and TNFR1, is presented in the Supplementary Materials (Supplementary File S1).

#### 2.5.2. PPI Network and Functional Enrichment Analysis

PPI network of the selected target proteins was analyzed using the STRING database (version 12.0, https://string-db.org, accessed on 7 April 2026)) [22]. Only interactions with high confidence scores (≥0.7) were included to construct the network. Topological properties—including the number of nodes, edges, and average node degree were calculated to assess network connectivity. The PPI enrichment *p*-value was used to determine whether the observed interactions were significantly more frequent than expected by chance. Functional enrichment analysis, including Gene Ontology (biological process [BP], molecular function [MF], cellular component [CC]) and KEGG and WikiPathways pathway analysis, was performed within STRING [22,23]. Terms with a false discovery rate (FDR) ≤ 0.05 were considered statistically significant. Network and enrichment results were visualized using STRING’s built-in tools.

#### 2.5.3. MCC-Based Hub Protein Identification

To identify the most central hub proteins within the SGLT2-associated PPI network, Maximal Clique Centrality (MCC) analysis was performed using Cytoscape (version 3.10.4) [24], with the CytoHubba plugin [25]. The MCC algorithm prioritizes nodes based on their involvement in maximal cliques, effectively highlighting proteins with critical topological importance. The top-ranked hub proteins identified by MCC were used for downstream functional interpretation and validation. The top eight proteins with the highest MCC scores were selected as core hub proteins for downstream interpretation.

## 3. Results

### 3.1. Baseline Characteristics

Among 1247 patients undergoing open heart surgery during the study period, 620 (49.7%) had T2DM and constituted the analytic cohort. Of these, 320 patients (51.6%) received chronic SGLT2 inhibitor therapy, whereas 300 patients (48.4%) were managed without SGLT2 inhibitors.

Baseline demographic and clinical characteristics are presented in Table 1. Overall, baseline characteristics were broadly comparable between groups and were further balanced after inverse probability of treatment weighting, with all standardized mean differences below 0.1. A modest difference in glycemic control was observed prior to weighting, with slightly lower HbA1c levels in the SGLT2 inhibitor group; this difference was attenuated after IPTW.

Before statistical adjustment, several variables reflected real-world treatment allocation patterns. Following inverse probability of treatment weighting, adequate covariate balance was achieved across all prespecified baseline characteristics.

### 3.2. Primary Outcome: First Rehospitalization

During the 12-month follow-up period, rehospitalization occurred significantly less frequently among patients receiving chronic SGLT2 inhibitor therapy compared with those managed without these agents.

To characterize the clinical drivers of rehospitalization, all events were systematically categorized into predefined phenotypic domains, including heart failure-related, arrhythmic, renal metabolic, pulmonary, ischemic, infectious, and surgery-related causes. The distribution of these phenotypes is summarized in Table 2.

Heart failure–related complications were the most frequently observed phenotype in both groups and occurred less frequently among patients receiving SGLT2 inhibitors. Similar trends were observed for pulmonary edema, atrial arrhythmias, electrolyte disturbances, and chronic obstructive pulmonary disease exacerbations.

Following this mechanistic characterization, the association between chronic SGLT2 inhibitor therapy and the risk of first rehospitalization was quantitatively evaluated using both time-to-event and recurrent event statistical frameworks. In unadjusted analyses, as well as after rigorous adjustment for potential confounding via inverse probability of treatment weighting, SGLT2 inhibitor use was associated with a lower risk of first rehospitalization within 12 months. These findings were consistent across multiple analytic approaches, including time-dependent Cox regression models accounting for delayed treatment initiation, Fine-Gray competing risk models considering death as a competing event, and recurrent-event models assessing cumulative rehospitalization burden (Table 3).

These analyses consistently showed lower rehospitalization rates among SGLT2 inhibitor users across time-to-event and recurrent-event models. Effect estimates are presented as hazard ratios (HRs) and incidence rate ratios (IRRs). The incidence of 12-month rehospitalization was 34.0% in the non-SGLT2 group and 21.9% in the SGLT2 group.

### 3.3. Competing Risk Analysis

When accounting for all-cause mortality as a competing event, Fine–Gray subdistribution hazard models showed a lower cumulative incidence of rehospitalization over 12 months among patients receiving chronic SGLT2 inhibitor therapy. The magnitude and direction of this association were consistent with those observed in time-dependent Cox regression analyses. Cumulative incidence function curves illustrated the separation between patients receiving SGLT2 inhibitors and those managed without these agents during follow-up.

### 3.4. Recurrent Rehospitalization Events

**Figure 2:**
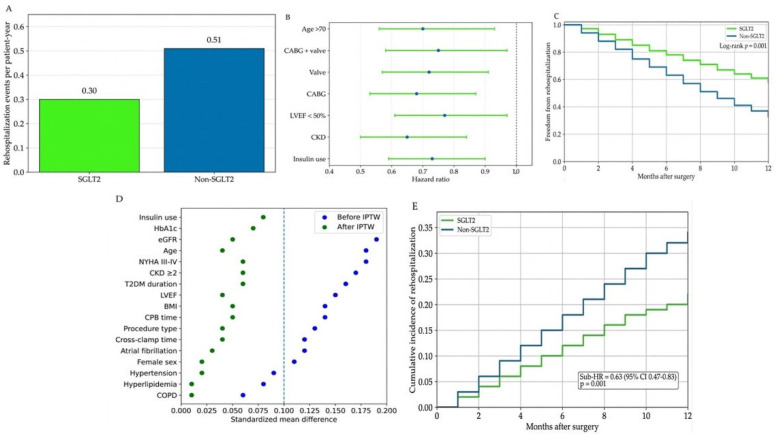
(**A**) Recurrent events analysis comparing incidence rate ratios between the SGLT2 and non–SGLT2 groups. (**B**) Subgroup analysis showing hazard ratios (HRs) with 95% confidence intervals (95% CIs). (**C**) Kaplan–Meier curves for freedom from rehospitalization after surgery in the SGLT2 versus non–SGLT2 groups. (**D**) Covariate balance before and after inverse probability of treatment weighting (IPTW), presented as standardized mean differences (SMDs). (**E**) Fine–Gray cumulative incidence curves for rehospitalization, accounting for death as a competing event. SGLT2, sodium–glucose cotransporter 2; HR, hazard ratio; 95% CI, 95% confidence interval; IPTW, inverse probability of treatment weighting; SMD, standardized mean difference.

Beyond the assessment of time to first rehospitalization, analyses incorporating negative binomial regression models were performed to evaluate cumulative rehospitalization burden. Using these models to account for multiple rehospitalization episodes per patient, SGLT2 inhibitor use was associated with a lower incidence rate of rehospitalization compared with non-users (Figure 2A).

### 3.5. Sensitivity and Subgroup Analyses

Prespecified sensitivity analyses showed similar associations between SGLT2 inhibitor use and rehospitalization risk across alternative modeling strategies and clinically relevant subgroups, including stratification by baseline cardiac and renal function (Figure 2B). No significant effect modification was observed in interaction-term analyses within IPTW-weighted Cox models.

### 3.6. Time-to-Event Sensitivity Analysis (Kaplan–Meier/Landmark Consistency)

Time-to-event sensitivity analyses showed patterns similar to those observed in the primary analysis. Kaplan–Meier curves demonstrated separation between treatment groups during the 12-month follow-up (Figure 2C).

After application of inverse probability of treatment weighting, a stabilized weighted pseudo-population of 618.4 was obtained. Covariate balance between treatment groups was evaluated using standardized mean differences, with values below 0.1 indicating adequate balance (Figure 2D).

Competing risk analyses using Fine–Gray subdistribution hazard models yielded results consistent with those obtained from the primary time-dependent Cox regression models. The cumulative incidence of rehospitalization remained lower among patients receiving SGLT2 inhibitor therapy after accounting for death as a competing event (Figure 2E).

### 3.7. In Silico Results

#### 3.7.1. Molecular Docking Results

Molecular docking analyses identified binding of both empagliflozin and dapagliflozin to SGLT2, involving amino acid residues within the transporter binding pocket (Table 4). Predicted binding energies, RMSD values, and hydrogen bond interactions for empagliflozin and dapagliflozin across investigated targets are summarized in Table 4.

Binding energies (kcal/mol) and RMSD values correspond to docking poses selected according to predefined criteria (lowest binding energy and RMSD ≤ 2 Å). Hydrogen bond interactions between ligands and amino acid residues within predicted binding pockets are listed in Table 4.

**Figure 3:**
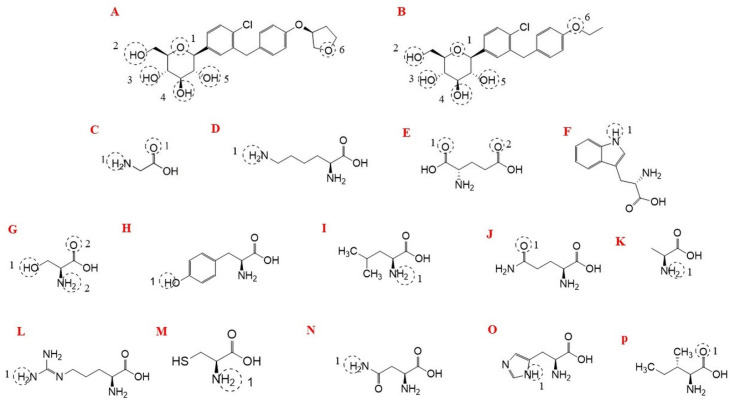
Molecular structures and amino acid residues involved in ligand–protein interactions. (**A**) Empagliflozin; (**B**) Dapagliflozin; (**C**) Glycine (Gly); (**D**) Lysine (Lys); (**E**) Glutamic acid (Glu); (**F**) Tryptophan (Trp); (**G**) Serine (Ser); (**H**) Tyrosine (Tyr); (**I**) Leucine (Leu); (**J**) Glutamine (Gln); (**K**) Alanine (Ala); (**L**) Arginine (Arg); (**M**) Cysteine (Cys); (**N**) Asparagine (Asn); (**O**) Histidine (His); (**P**) Isoleucine (Ile).

Interactions were observed between both compounds and NHE1, AMPK, and PPAR-α/γ. Additional interactions were identified with inflammatory signaling proteins, including IKKβ, NLRP3, IL-6Rα, and TNFR1. A two-dimensional (2D) schematic representation illustrating interaction sites and amino acid residues involved in ligand–protein complexes is presented in Figure 3.

**Figure 4:**
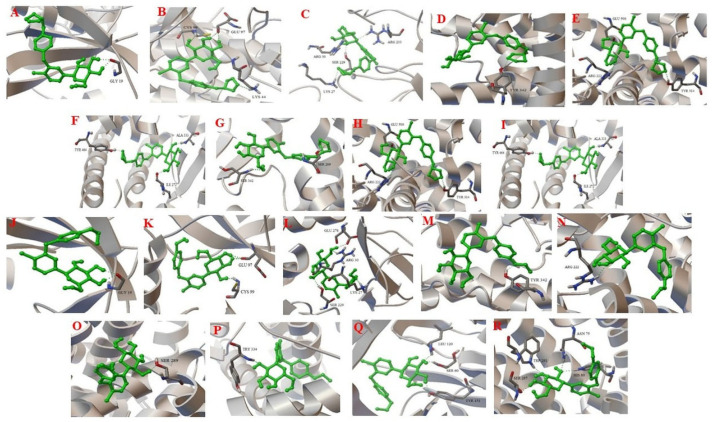
Molecular docking analysis of SGLT2 inhibitors showing three-dimensional protein–ligand complexes and interacting amino acid residues of empagliflozin and dapagliflozin with their target proteins. Empagliflozin complexes include (**A**) Empagliflozin-AMPK, (**B**) Empagliflozin-IKKβ, (**C**) Empagliflozin-IL-6Rα, (**D**) Empagliflozin-NHE1, (**E**) Empagliflozin-NLRP3, (**F**) Empagliflozin-PPAR-α, (**G**) Empagliflozin-PPAR-γ, (**H**) Empagliflozin-TNFR1, and (**I**) Empagliflozin-SGLT2. Dapagliflozin complexes include (**J**) Dapagliflozin-AMPK, (**K**) Dapagliflozin-IKKβ, (**L**) Dapagliflozin-IL-6Rα, (**M**) Dapagliflozin-NHE1, (**N**) Dapagliflozin-NLRP3, (**O**) Dapagliflozin-PPAR-α, (**P**) Dapagliflozin-PPAR-γ, (**Q**) Dapagliflozin-TNFR1, and (**R**) Dapagliflozin-SGLT2.

Representative 3D protein–ligand complexes, highlighting the interacting residues of empagliflozin and dapagliflozin with their respective targets (SGLT2, NHE1, AMPK, and inflammatory mediators), are shown in Figure 4.

#### 3.7.2. PPI Network Topology

**Figure 5:**
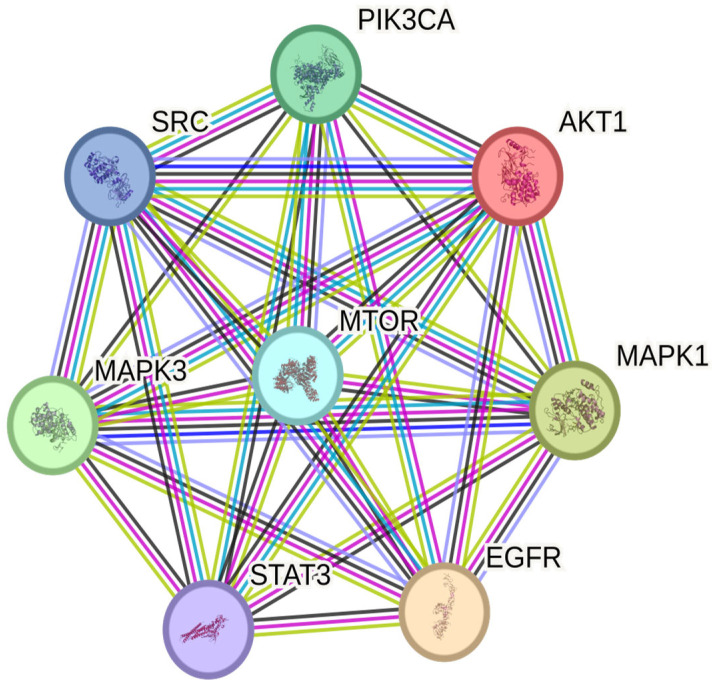
PPI network of SGLT2-associated proteins constructed using STRING (v12.0). Nodes represent proteins (AKT1, EGFR, MAPK1, MAPK3, MTOR, PIK3CA, SRC, STAT3) and edges denote interactions, with thickness reflecting interaction confidence.

The PPI network of SGLT2-associated proteins was constructed using the STRING database (v12.0). Subsequent MCC-based topological analysis in Cytoscape identified a densely interconnected core subnetwork composed of eight proteins (AKT1, EGFR, MAPK1, MAPK3, MTOR, PIK3CA, SRC, and STAT3), forming a fully connected complete network (K8, 28 edges) (Figure 5).

The network showed an average node degree of 7 and a PPI enrichment *p*-value of 0.0106.

##### Functional Enrichment Analysis

Functional enrichment analysis of the SGLT2-associated PPI network identified enrichment across biological processes (BP), molecular functions (MF), cellular components (CC), and signaling pathways (Table 5 and Table 6). Molecular functions included protein kinase activity and phosphoprotein binding. KEGG and WikiPathways analyses included EGFR tyrosine kinase inhibitor resistance and PI3K/AKT signaling pathways.

##### MCC-Based Hub Protein Identification

**Figure 6:**
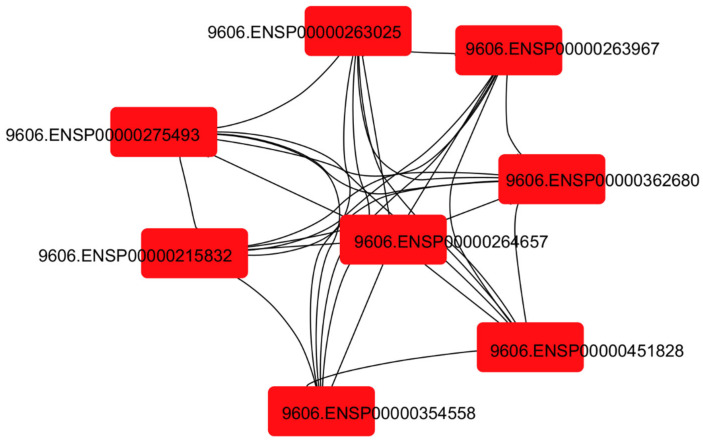
MCC-based topological analysis of the SGLT2-associated hub interactome. The network illustrates the topologically central signaling proteins identified through the Maximal Clique Centrality (MCC) algorithm. The STRING IDs displayed within the nodes correspond to the following hub proteins: 9606.ENSP00000263025 (AKT1), 9606.ENSP00000263967 (PIK3CA), 9606.ENSP00000275493 (EGFR), 9606.ENSP00000362680 (SRC), 9606.ENSP00000264657 (STAT3), 9606.ENSP00000215832 (MTOR), 9606.ENSP00000451828 (MAPK1), 9606.ENSP00000354558 (MAPK3). Hub proteins identified by MCC analysis are highlighted in red.Dense interconnectivity between nodes reflects network connectivity within the identified kinase-related signaling proteins.

Network analysis highlighted a compact signaling module composed of eight highly interconnected proteins (AKT1, EGFR, MAPK1, MAPK3, MTOR, PIK3CA, SRC, and STAT3) (Figure 6, Table 7).

## 4. Discussion

This study found that chronic SGLT2 inhibitor therapy was statistically significantly associated with a lower risk of first rehospitalization and reduced cumulative 12-month event burden in patients with T2DM undergoing cardiac surgery. Our findings demonstrate a consistent association across multiple analytical frameworks, including inverse probability of treatment weighting and recurrent-event modeling, addressing an important evidence gap in the current perioperative literature [5,11].

In absolute terms, the incidence of 12-month rehospitalization was reduced from 34.0% in the non-SGLT2 group to 21.9% in the SGLT2 group. For contextual reference, the observed absolute risk difference corresponds to an approximate number needed to be associated with benefit in 8 patients to prevent one rehospitalization event within 12 months, acknowledging that this estimate is derived from an observational cohort and does not imply a causal treatment effect.

Kaplan–Meier analyses demonstrated early and sustained separation between treatment groups. The persistence of curve separation supports a durable reduction in rehospitalization risk over time and argues against a short-term or peri-discharge-restricted effect.

In addition to the favorable clinical outcomes observed in this cohort, in silico analyses provide a mechanistic context by delineating a densely interconnected protein interaction network dominated by kinase-regulated survival signaling pathways, including AKT1, MTOR, and STAT3, together with key nodes involved in inflammatory regulation and cellular stress responses. Docking simulations further suggested differential interaction patterns, including variation in predicted binding affinity toward NHE1 between dapagliflozin and empagliflozin. These findings indicate that although SGLT2 inhibitors share a common primary pharmacological class effect, their broader cardiorenal stabilizing properties may reflect heterogeneity in secondary molecular interaction profiles across individual agents [6,9]. Such observations support the concept that SGLT2 inhibition extends beyond conventional glycemic regulation and may represent a multidimensional pharmacological strategy relevant to the physiological and metabolic stress associated with cardiac surgery [7,8]. However, these mechanistic interpretations should be considered exploratory, and the observational design of the clinical component limits causal inference.

The observed reduction in both first rehospitalization (HR 0.64) and cumulative event burden (IRR 0.61) is particularly notable when considered alongside results from landmark SGLT2 inhibitor trials. Large-scale studies such as EMPA-REG OUTCOME and DAPA-HF have demonstrated significant cardiorenal benefits in ambulatory diabetic and heart failure populations [5,7]. The present findings extend these observations to the perioperative environment of cardiac surgery, a setting characterized by acute physiological stress and complex metabolic perturbations. In this context, the magnitude of association observed in the current cohort appears numerically greater than that typically reported in general diabetic populations, potentially reflecting the amplified clinical relevance of SGLT2 inhibition during periods of intense neurohormonal activation and systemic inflammatory response [2,6].

Furthermore, by employing rigorous statistical frameworks similar to those advocated by Tallarico et al., we were able to account for the ‘noise’ of early postoperative mortality and confounding by indication, which may have masked these benefits in previous retrospective analyses [11]. This observation is consistent with the possibility that SGLT2 inhibitors may exert effects beyond glucose lowering, including potential perioperative cardiorenal stabilization, possibly related to hemodynamic shifts and metabolic substrate utilization that characterize the vulnerable post-cardiac surgery phenotype [8,26].

While the cardiometabolic benefits of SGLT2 inhibitors are increasingly recognized, caution remains warranted in vulnerable perioperative settings. Patients with acute decompensated heart failure, hemodynamic instability, or systemic illness may exhibit altered risk-benefit profiles, necessitating individualized perioperative decision-making [27,28]. In addition, emerging perioperative literature has highlighted the potential risk of euglycemic diabetic ketoacidosis and the importance of temporary discontinuation and careful metabolic monitoring in selected surgical patients [28,29,30]. Accordingly, careful patient selection, individualized perioperative risk assessment, and context-specific management strategies should be considered, particularly in high-risk surgical populations.

Postoperative rehospitalizations were predominantly driven by hemodynamic decompensation and cardiorenal dysfunction rather than surgical complications, highlighting the vulnerability of T2DM patients to perioperative metabolic and inflammatory stress. In this high-risk context, traditional glycemic control may play a secondary role compared with the need for aggressive cardiorenal stabilization [26,27,28,29,30,31,32,33].

A central finding of our in silico analysis is the interaction between SGLT2 inhibitors and NHE1. Myocardial NHE1 overactivity is a hallmark of the ‘vulnerable phenotype’ following cardiac surgery, leading to intracellular sodium and calcium overload, thereby exacerbating oxidative stress and contractile dysfunction [6,34]. While SGLT2 inhibitors are often discussed as a class, docking simulations revealed an approximately 4 kcal/mol difference in predicted binding affinity toward NHE1, with dapagliflozin demonstrating stronger modeled interaction than empagliflozin. From a mechanistic perspective, this difference may indicate heterogeneity in potential modulation of myocardial ion homeostasis, although docking-derived binding energies primarily reflect relative interaction stability under simplified computational conditions rather than absolute pharmacodynamic potency. This observation suggests that while both agents may contribute to systemic cardiorenal protection through SGLT2 inhibition, their relative contributions to myocardial resilience may differ. Such heterogeneous molecular interaction profiles may partly contribute to the clinical associations observed in the perioperative setting [34,35,36].

The molecular docking analyses provide a quantitative indication of these interactions, demonstrating high-affinity binding of empagliflozin and dapagliflozin to SGLT2 (−15.39 and −15.37 kcal/mol, respectively) and critical secondary targets. Notably, the interaction with NHE1 showed a marked difference in affinity (−6.24 kcal/mol for empagliflozin vs. –10.02 kcal/mol for dapagliflozin), reinforcing the potential for drug-specific modulation of ionic homeostasis. Beyond ion exchange, both compounds engaged metabolic and inflammatory regulators, including AMPK (–9.94 and –8.53 kcal/mol), PPAR-α (−12.15 and −9.66 kcal/mol), PPAR-γ (−11.96 and −11.63 kcal/mol), NLRP3 (−11.89 and −10.54 kcal/mol), and IKKβ (−12.83 and −11.17 kcal/mol). The identification of short-range hydrogen bonding with key residues—including ASN75, TRP291, and GLN457 for SGLT2; ARG222 and TYR314 for NLRP3; and ALA333, TYR464, and ILE272 for PPAR-α—supports the preservation of classical on-target pharmacodynamics while providing mechanistic plausibility for downstream cardiorenal, metabolic, and anti-inflammatory effects [35,36,37,38,39,40]. These molecular interaction patterns suggest that SGLT2 inhibitors may influence multiple cardiorenal regulatory pathways in the high-stress surgical environment, where simultaneous modulation of ion exchange and inflammatory signaling may contribute to postoperative stability [34,38].

The translational relevance of these molecular interactions may be supported by established physiological mechanisms.

High-affinity SGLT2 binding is associated with natriuresis and osmotic diuresis, potentially attenuating perioperative fluid overload and ventricular wall stress, which are critical triggers for rehospitalization in the early postoperative phase [5,8]. Furthermore, the potent NHE1 modulation we identified—particularly the higher affinity profile of dapagliflozin—may reduce cytosolic Na^+^ accumulation and secondary Ca^2+^ overload, thereby improving myocardial contractility and mitigating ischemia–reperfusion injury [35,36]. The simultaneous engagement of AMPK and PPAR suggests an enhancement of myocardial energy efficiency and metabolic substrate utilization, potentially favoring mitochondrial resilience. This is highly consistent with observed improvements in cardiac energetics following empagliflozin therapy in experimental models [32,33]. Finally, anti-inflammatory interactions suggested by our docking data may be related to reduced NLRP3 inflammasome activation and NF-κB signaling, processes implicated in postoperative myocardial and renal injury [39,40].

Collectively, these pathways form a coherent mechanistic framework that may contribute to the cardiorenal resilience observed in our cohort. Mechanistic convergence between molecular docking and clinical outcomes may support a coherent translational framework in our study. The marked reductions in both first and recurrent rehospitalizations, primarily driven by heart failure exacerbation and cardiorenal decompensation, appear to align closely with the predicted effects of SGLT2 inhibition on fluid balance, myocardial energy metabolism, intracellular ion homeostasis, and inflammatory modulation. However, a nuanced interpretation is required; while docking affinities provide a strong indicator of mechanistic plausibility and target engagement, they do not directly quantify in vivo inhibitory potency or systemic pharmacodynamic activity. Factors such as drug bioavailability, tissue-specific distribution, and local concentrations during the perioperative period remain critical variables that complement these in silico predictions. Nonetheless, the high binding stability observed across multiple cardiorenal targets suggests that SGLT2 inhibitors may provide a potential molecular baseline for clinical resilience following cardiac surgery.

The application of IPTW within our analytical framework was designed to minimize selection bias and confounding, as reflected by improved balance across covariates (SMD < 0.1). Furthermore, adjustment for immortal time bias and competing mortality strengthened the temporal association between SGLT2 inhibitor exposure and postoperative outcomes. This methodological rigor supports the internal consistency of the observed associations while acknowledging the limitations inherent to observational study designs [11].

Clinically, these findings suggest that structured perioperative SGLT2 inhibitor management may be explored within standardized perioperative management frameworks, including ERAS pathways, in carefully selected patients. However, such implementation must be performed under vigilant monitoring for metabolic stability and the potential risk of euglycemic ketoacidosis. Ultimately, recognizing SGLT2 inhibitors as potential perioperative cardiorenal modulators, rather than solely anti-hyperglycemic agents, may offer an evolving clinical perspective [5,8]

From a surgical quality perspective, rehospitalizations in our cohort appear to reflect modifiable vulnerabilities of the cardiorenal axis rather than unavoidable procedural complications. The perioperative period represents a convergence of metabolic, inflammatory, and hemodynamic stressors exacerbated by cardiopulmonary bypass and T2DM-associated endothelial dysfunction. Through potential effects on ion homeostasis, improving myocardial energy efficiency, and attenuating inflammatory signaling, SGLT2 inhibitors may contribute to more favorable postoperative trajectories and a reduced cumulative readmission burden.

Several limitations should be acknowledged. The network pharmacology findings were not externally validated and should therefore be interpreted as exploratory. The lack of consistent perioperative biomarker data precluded in vivo confirmation of mechanistic pathways, rendering the in silico PPI, docking, and functional enrichment analyses hypothesis-generating rather than confirmatory. Additionally, the relatively modest sample size may have limited detection of rare but serious complications, including euglycemic ketoacidosis. Finally, conclusions regarding isolated mortality should be interpreted cautiously, given the exploratory nature of these secondary analyses. Collectively, these considerations highlight the need for prospective, multicenter studies to validate both clinical outcomes and mechanistic hypotheses. Residual and unmeasured confounding may persist despite statistical adjustment. Factors such as frailty status, perioperative hemodynamic management, nutritional condition, and clinician-driven prescribing decisions may have influenced both treatment allocation and outcomes. Furthermore, the single-center retrospective design introduces potential selection bias and may limit the generalizability of the findings to other institutions with different perioperative management protocols. These limitations should be considered when interpreting the observed associations. In clinical practice, this typically involves temporary discontinuation of SGLT2 inhibitors prior to surgery and cautious reinitiation after postoperative stabilization. Therefore, potential benefits in reducing rehospitalization must be interpreted alongside established perioperative safety considerations, emphasizing the need for individualized management strategies.

In addition, differences in healthcare systems, surgical practices, and perioperative management protocols may influence the applicability of these findings. Therefore, validation in multicenter cohorts and prospective studies is warranted to further assess generalizability across diverse clinical settings. Formal prespecified subgroup analyses were limited, and exploratory stratified assessments should therefore be interpreted cautiously.

Beyond individual protein–ligand interactions, PPI and functional enrichment analyses highlight eight central hub proteins—AKT1, mTOR, STAT3, EGFR, PIK3CA, SRC, MAPK1, and MAPK3—that coordinate kinase-driven survival, metabolic adaptation, and stress-response pathways. GO/KEGG enrichment suggested involvement of PI3K/AKT, MAPK/ERK, and ErbB signaling, potentially associated with myocardial resilience under ischemic and inflammatory stress. Collectively, these network and docking findings are consistent with biologically plausible mechanisms for the observed reduction in postoperative rehospitalization associated with chronic SGLT2 inhibitor therapy.

These mechanistic findings should be interpreted as hypothesis-generating and do not establish causal relationships but rather provide biological plausibility supporting the observed clinical associations. As a single-center retrospective observational study, causal inference is inherently limited, and the findings should therefore be interpreted as associative despite the application of advanced statistical adjustment methods.

This network-based analysis should be interpreted as a mechanistic hypothesis-generating framework rather than direct molecular validation. High-affinity docking to SGLT2, NHE1, AMPK, and NLRP3, together with hub protein connectivity, may offer a potential mechanistic explanation for the observed reductions in both first and recurrent rehospitalizations. In line with institutional perioperative protocols, SGLT2 inhibitors were temporarily discontinued preoperatively and resumed postoperatively once hemodynamic stability was achieved, with careful monitoring for euglycemic ketoacidosis, volume depletion, and metabolic derangements. The strong predicted TNFR1 binding should be interpreted cautiously, as docking scores may also reflect structural complementarity within the modeled binding pocket rather than direct pharmacological engagement under physiological conditions.

An important methodological consideration concerns the potential influence of baseline glycemic control on postoperative outcomes. To minimize this source of confounding, HbA1c was explicitly incorporated into the propensity score model used for inverse probability of treatment weighting, thereby accounting for baseline differences in metabolic status between treatment groups. Notably, the association between SGLT2 inhibitor therapy and reduced rehospitalization persisted after IPTW adjustment, suggesting that the observed association cannot be explained solely by improved glycemic control. Instead, these findings suggest that the observed associations with SGLT2 inhibition may reflect broader cardiometabolic mechanisms that become particularly relevant in the physiologically stressed postoperative environment. In this context, the convergence between the clinical outcome analyses and the molecular interaction patterns identified in the docking experiments may provide a biological framework linking metabolic modulation, myocardial energy homeostasis, and inflammatory regulation to improved postoperative trajectories. This integrated interpretation is consistent with the overall pattern of the study findings and provides a conceptual framework for interpreting the observed associations.

Taken together, the integration of advanced causal inference methods with hypothesis-generating in silico network pharmacology may offer a multidimensional framework for interpreting the potential perioperative benefits of SGLT2 inhibition.

From a translational perspective, these findings may help inform a structured perioperative approach to SGLT2 inhibitor management. Careful patient selection, temporary discontinuation prior to surgery, and reinitiation after hemodynamic and metabolic stabilization may help balance potential benefits and risks. Individualized perioperative risk assessment, particularly in patients with complex cardiometabolic profiles, may further optimize outcomes. These considerations should be interpreted cautiously and require validation in prospective clinical studies.

## 5. Conclusions

Chronic SGLT2 inhibitor therapy was associated with more favorable postoperative cardiometabolic outcomes in patients with T2DM undergoing cardiac surgery, including a lower risk of rehospitalization during follow-up. These findings should be interpreted cautiously, given the observational study design and the potential for residual confounding despite advanced statistical adjustment.

The integration of clinical analyses with exploratory in silico approaches may provide a mechanistic context supporting the observed associations rather than direct causal confirmation. The observed associations may relate to broader biological mechanisms involving modulation of myocardial ion homeostasis, cellular energy metabolism, and inflammatory signaling pathways.

Consistency across bias-aware analytical approaches, including IPTW adjustment, time-dependent exposure modeling, competing risk analysis, and recurrent-event frameworks, supports the internal coherence of the findings. However, these results remain hypothesis-generating. Prospective multicenter studies are required to further evaluate these observations, clarify perioperative management strategies, and establish standardized safety monitoring.

In summary, chronic SGLT2 inhibitor therapy was associated with reduced postoperative rehospitalization in patients with T2DM undergoing cardiac surgery, but prospective validation is required before clinical recommendations can be made.

## Figures and Tables

**Table 1:** Baseline Demographic and Clinical Characteristics of the Study Population.

Variable	SGLT2 (*n* = 320)	Non-SGLT2 (*n* = 300)	*p*-Value
Age, years	66.1 ± 7.9	66.8 ± 8.3	0.28
Female sex, *n* (%)	111 (34.7)	109 (36.3)	0.68
BMI, kg/m^2^	29.7 ± 4.1	30.1 ± 4.3	0.24
Duration of T2DM, years	9.7 ± 6.4	10.1 ± 6.7	0.46
HbA1c, %	7.9 ± 1.0	8.3 ± 1.2	0.041
Insulin use, *n* (%)	130 (40.6)	138 (46.0)	0.18
Hypertension, *n* (%)	258 (80.6)	235 (78.3)	0.48
Hyperlipidemia, *n* (%)	243 (75.9)	224 (74.7)	0.73
Chronic kidney disease, *n* (%)	89 (27.8)	87 (29.0)	0.73
COPD, *n* (%)	33 (10.3)	32 (10.7)	0.87
Atrial fibrillation, *n* (%)	46 (14.4)	43 (14.3)	0.97
Peripheral arterial disease, *n* (%)	28 (8.8)	27 (9.0)	0.92
History of stroke/TIA, *n* (%)	19 (5.9)	18 (6.0)	0.95
LVEF, %	50.8 ± 8.7	49.9 ± 9.0	0.28
NYHA class III–IV, *n* (%)	87 (27.2)	84 (28.0)	0.82
Pulmonary hypertension, *n* (%)	51 (15.9)	49 (16.3)	0.89

Values are presented as mean ± standard deviation, median (interquartile range), or number (percentage), as appropriate. Covariate balance after inverse probability of treatment weighting was assessed using standardized mean differences. BMI = body mass index; COPD = chronic obstructive pulmonary disease; LVEF = left ventricular ejection fraction; NYHA = New York Heart Association; TIA = transient ischemic attack; T2DM = type 2 diabetes mellitus.

**Table 2:** Clinical Complications of Rehospitalization After Cardiac Surgery According to SGLT2 Inhibitor Use.

Rehospitalization Phenotype	SGLT2 (*n* = 70)	Non–SGLT2 (*n* = 102)	*p*-Value
Heart failure–related complications			
Worsening heart failure/congestion	22 (31.4%)	46 (45.1%)	0.048
Low cardiac output syndrome (Late)	1 (1.4%)	9 (8.8%)	0.027
Pulmonary edema	3 (4.3%)	14 (13.7%)	0.030
Renal and metabolic complications			
Renal decompensation/AKI progression	13 (18.6%)	14 (13.7%)	0.47
Volume depletion/hypotension	3 (4.3%)	8 (7.8%)	0.34
Electrolyte disturbances	1 (1.4%)	10 (9.8%)	0.020
Arrhythmic complications			
Atrial fibrillation/flutter	2 (2.9%)	12 (11.8%)	0.023
Ventricular arrhythmias	1 (1.4%)	3 (2.9%)	0.52
Bradyarrhythmia/conduction disorder	2 (2.9%)	4 (3.9%)	0.72
Pulmonary complications			
Respiratory failure	4 (5.7%)	4 (3.9%)	0.58
Pneumonia	3 (4.3%)	6 (5.9%)	0.64
Exacerbation of COPD	0 (0%)	6 (5.9%)	0.038
Ischemic and graft-related complications			
Acute coronary syndrome/graft dysfunction	2 (2.9%)	5 (4.9%)	0.51
Recurrent angina without infarction	3 (4.3%)	6 (5.9%)	0.64
Infectious and surgical-site phenotypes			
Sternal wound infection	2 (2.9%)	3 (2.9%)	0.99
Superficial surgical-site infection	3 (4.3%)	5 (4.9%)	0.85
Mediastinitis	0 (0%)	1 (1.0%)	—
Neurological and thromboembolic complications			
Transient ischemic attack	1 (1.4%)	2 (2.0%)	0.76
Ischemic stroke	1 (1.4%)	3 (2.9%)	0.52
Pulmonary embolism	1 (1.4%)	2 (2.0%)	0.76
Other cardiac/surgical causes			
Postoperative bleeding/Late hemothorax	1 (1.4%)	2 (2.0%)	0.76
Prosthetic valve–related complication	1 (1.4%)	2 (2.0%)	0.76
Unspecified cardiac/surgical cause	2 (2.9%)	3 (2.9%)	0.99

**Table 3:** Clinical Outcomes and Endpoint Events.

Variable	SGLT2 (*n* = 320)	Non-SGLT2 (*n* = 300)	*p*-Value
Primary and secondary endpoints			
90-day rehospitalization, *n* (%)	32 (10.0%)	60 (20.0%)	0.002
12-month rehospitalization, *n* (%)	70 (21.9%)	102 (34.0%)	0.001
Cardiovascular death, *n* (%)	8 (2.5%)	11 (3.7%)	0.38
Composite endpoint (rehospitalization + CV death), *n* (%)	78 (24.4%)	113 (37.7%)	0.001
Time-to-event analyses			
Primary HR (12-month rehospitalization)	HR = 0.64 (95% CI 0.48–0.85)		0.002
Composite HR (rehospitalization + CV death)	HR = 0.62 (95% CI 0.47–0.83)		0.001
Recurrent event analyses			
Recurrent rehospitalization events, total	95	153	0.002
Rehospitalization burden (events per patient-year)	0.30	0.51	0.003
Recurrent-event IRR	IRR = 0.61 (95% CI 0.46–0.82)		0.001

CV = cardiovascular; HR = hazard ratio; IRR = incidence rate ratio. *p*-values for binary endpoints (90-day and 12-month rehospitalization, cardiovascular death, and composite endpoint) were obtained using the χ^2^ test. Hazard ratios and 95% CI for time-to-event outcomes were estimated using time-dependent Cox proportional hazards regression with IPTW. IRRs and *p*-values for recurrent event counts and rehospitalization burden were derived from negative binomial regression models with log-transformed follow-up time as an offset and robust variance estimators to account for within-patient correlation. All effect estimates reflect comparisons between chronic SGLT2 inhibitor users and non-users.

**Table 4:** Molecular docking results of empagliflozin and dapagliflozin against selected cardiometabolic and inflammatory targets.

Compound	Target Protein	Energy Score (kcal/mol)	RMSD (Å)	H-Bond (Distance Å)	PDB ID
Empagliflozin	AMPK	−9.94	0.72	H-3 with O-1 of GLY 19 (1.855); H-3 with O-2 of GLY 19 (1.793)	6B2E
Empagliflozin	IKKβ	−12.83	0.33	O-6 with H-1 of LYS 44 (2.168); O-5 with H-1 of CYS 99 (2.196); H-3 with O-1 of GLU 97 (1.85); H-4 with O-1 of GLU 97 (1.827)	4KIK
Empagliflozin	IL-6Rα	−9.56	0.77	O-5 with H-1 of SER 229 (1.831); O-2 with H-1 of ARG 233 (2.164); O-6 with H-1 of LYS 27 (2.221); O-1 with H-1 of ARG 30 (1.81)	1P9M
Empagliflozin	NHE1	−6.24	0.50	O-6 with H-1 of TYR 342 (1.658)	7DSX
Empagliflozin	NLRP3	−11.89	0.46	O-4 with H-1 of ARG 222 (2.136); O-6 with H-1 of TYR 314 (2.018); H-2 with O-2 of GLU 500 (2.236)	8RI2
Empagliflozin	PPAR-α	−12.15	0.92	O-5 with H-1 of ALA 333 (2.214); O-6 with H-1 of TYR 464 (2.079); H-2 with O-1 of ILE 272 (1.937)	7BQ3
Empagliflozin	PPAR-γ	−11.96	1.12	O-4 with H-1 of SER 289 (1.97); O-2 with H-2 of SER 342 (2.154)	6MD4
Empagliflozin	TNFR1	−14.93	1.92	O-2 with H-1 of SER 60 (2.245); H-2 with O-2 of SER 60 (1.807); O-2 with H-1 of LEU 120 (2.089)	7KPB
Empagliflozin	SGLT2	−15.39	0.92	O-5 with H-1 of ASN 75 (1.786); O-4 with H-1 of TRP 291 (2.191); H-2 with O-1 of GLN 457 (2.176)	7VSI
Dapagliflozin	AMPK	−8.53	0.37	H-3 with O-1 of GLY 19 (2.027); H-4 with O-1 of GLY 19 (1.92)	6B2E
Dapagliflozin	IKKβ	−11.17	0.33	O-5 with H-1 of CYS 99 (2.238); H-4 with O-1 of GLU 97 (1.996); H-3 with O-1 of GLU 97 (1.94)	4KIK
Dapagliflozin	IL-6Rα	−9.96	0.41	O-5 with H-1 of SER 229 (1.913); O-6 with H-1 of LYS 27 (1.926); O-2 with H-1 of ARG 30 (1.976); H-2 with O-2 of GLU 278 (2.083)	1P9M
Dapagliflozin	NHE1	−10.02	0.53	O-6 with H-1 of TYR 342 (1.907)	7DSX
Dapagliflozin	NLRP3	−10.54	0.61	O-4 with H-1 of ARG 222 (2.181)	8RI2
Dapagliflozin	PPAR-α	−9.66	0.65	O-5 with H-1 of TYR 334 (2.197)	7BQ3
Dapagliflozin	PPAR-γ	−11.63	0.42	H-5 with O-1 of SER 289 (2.028)	6MD4
Dapagliflozin	TNFR1	−14.87	1.42	O-4 with H-2 of SER 60 (2.046); O-4 with H-1 of LEU 120 (2.122); H-2 with O-1 of TYR 151 (1.971); H-? with O-1 of TYR 151 (2.227)	7KPB
Dapagliflozin	SGLT2	−15.37	0.76	O-5 with H-1 of ASN 75 (1.92); O-5 with H-1 of HIS 80 (2.075); O-4 with H-1 of TRP 291 (2.158); H-3 with O-2 of SER 287 (1.738)	8HEZ

**Table 5:** Hub proteins identified from the SGLT2-associated PPI network using MCC analysis.

Rank	Protein Symbol	Description	Degree (Number of Interactions)
1	SRC	Proto-oncogene tyrosine-protein kinase Src, a non-receptor tyrosine kinase involved in signal transduction	7
2	AKT1	RAC-alpha serine/threonine-protein kinase, regulates metabolism, proliferation, and survival	7
3	STAT3	Signal transducer and activator of transcription 3, mediates cellular responses to cytokines	7
4	EGFR	Epidermal growth factor receptor, receptor tyrosine kinase mediating cell growth and survival	7
5	PIK3CA	Catalytic subunit of phosphoinositide-3-kinase, involved in cell growth signaling	7
6	MTOR	Serine/threonine-protein kinase mTOR, central regulator of metabolism and growth	7
7	MAPK1	Mitogen-activated protein kinase 1, part of the MAPK/ERK signaling cascade	7
8	MAPK3	Mitogen-activated protein kinase 3, works with MAPK1 in the MAPK/ERK cascade	7

**Table 6:** Significantly enriched biological processes (BP), molecular functions (MF), cellular components (CC), and pathways for SGLT2-associated proteins. Count represents the number of proteins associated with each term, Strength indicates enrichment magnitude, and FDR is the false discovery rate-adjusted *p*-value.

Category	Term	Count (in Network)	Strength	FDR
BP	ERBB signaling pathway	5	3.85	8.50 × 10^−8^
BP	Cellular response to chemical stress	7	3.34	8.66 × 10^−9^
BP	Response to epidermal growth factor	4	3.28	1.38 × 10^−6^
MF	Protein kinase activity	7	2.10	8.00 × 10^−7^
MF	Phosphoprotein binding	4	2.41	3.00 × 10^−5^
MF	Protein serine/threonine kinase activity	6	2.07	3.98 × 10^−6^
CC	TOR complex	4	3.67	5.23 × 10^−7^
CC	PTEN phosphatase complex	3	3.39	2.83 × 10^−6^
Pathway	EGFR tyrosine kinase inhibitor resistance	8	8.19	2.87 × 10^−17^
Pathway	ErbB signaling pathway	7	6.80	1.86 × 10^−14^

**Table 7:** Core proteins identified by MCC-based network analysis.

Rank	Protein Symbol	Description	Degree	Betweenness Centrality	Closeness Centrality
1	EGFR	Epidermal growth factor receptor	7	0.0	1.0
2	PIK3CA	Phosphatidylinositol 3-kinase catalytic subunit alpha	7	0.0	1.0
3	MAPK3	Mitogen-activated protein kinase 3	7	0.0	1.0
4	SRC	Proto-oncogene tyrosine-protein kinase Src	7	0.0	1.0
5	MAPK1	Mitogen-activated protein kinase 1	7	0.0	1.0
6	AKT1	RAC-alpha serine/threonine-protein kinase	7	0.0	1.0
7	MTOR	Serine/threonine-protein kinase mTOR	7	0.0	1.0
8	STAT3	Signal transducer and activator of transcription 3	7	0.0	1.0

## Data Availability

The data presented in this study are available in this article and in the Supplementary Materials. Further inquiries can be directed to the corresponding author.

## Supplementary Materials

The following supporting information can be downloaded at: https://www.mdpi.com/article/10.3390/jcm15082873/s1, Supplementary File S1: Detailed Molecular Docking Workflow and Computational Validation Framework.

## Abbreviations

The following abbreviations are used in this manuscript:

AMPK	AMP-activated protein kinase
CABG	Coronary artery bypass graft surgery
ERAS	Enhanced recovery after surgery
GO	Gene Ontology
IKKβ	IκB kinase beta
IL-6Rα	Interleukin-6 receptor alpha
IPTW	Inverse probability of treatment weighting
IRR	Incidence rate ratio
KEGG	Kyoto Encyclopedia of Genes and Genomes
MCC	Maximal Clique Centrality
NHE1	Sodium–hydrogen exchanger 1
NLRP3	NOD-, LRR-, and pyrin domain-containing protein 3
PPI	Protein–protein interaction
PPAR-α/γ	Peroxisome proliferator-activated receptor alpha/gamma
RMSD	Root-mean-square deviation
SGLT2	Sodium–glucose cotransporter 2
SMD	Standardized mean difference
T2DM	Type 2 diabetes mellitus
TNFR1	Tumor necrosis factor receptor 1
