# Supplementary material for: SGLT2 Inhibition as a Perioperative Cardiorenal Stabilizer in Cardiac Surgery: Integrated Clinical Cohort and Pleiotropic Network-Based Pharmacological Analysis
Source: J Clin Med. 2026 Apr 10;15(8):2873. doi: 10.3390/jcm15082873 (PMC13115701; doi:10.3390/jcm15082873)
Supplement: Supplementary file 1 [file jcm-15-02873-s001.zip › jcm-4224608-supplementary.pdf]

Supplementary Materials Overview

This Supplementary Materials document provides detailed methodological descriptions, validation considerations, and transparency statements supporting the computational analyses presented in the main manuscript. The information included herein is intended to enhance reproducibility, clarify analytical decision frameworks, and provide additional structural and pharmacological context for the reported in silico findings without extending interpretative claims beyond those justified by the primary results. All supplementary content is aligned with the experimental objectives and reporting standards described in the main article and is provided to facilitate independent evaluation and replication of the computational workflow. The supplementary analyses are intended to enhance methodological transparency and should not be interpreted as independent mechanistic validation.

Supplementary File

Detailed Molecular Docking Workflow

This supplementary document provides a complete and reproducible description of the molecular docking workflow referenced in the main manuscript: SGLT2 Inhibition as a Perioperative Cardiorenal Stabilizer in Cardiac Surgery: Integrated Clinical Cohort and Pleiotropic Network-Based Pharmacological Analysis. The objective of these analyses was exploratory-to evaluate mechanistic plausibility rather than establish causality-by assessing potential interactions of empagliflozin and dapagliflozin with selected cardiometabolic and inflammatory targets.

1. Software Environment

All molecular docking analyses were performed using:

- AutoDock 4.2.6 for docking simulations
- AutoDockTools (MGLTools) version 1.5.6 for receptor–ligand preparation and grid definition
- Open Babel version 3.1.1 for file conversion
- ChemDraw Ultra 12.0 and ChemBio3D Ultra 13.0 for ligand construction and energy minimization
- Python Molecular Viewer (PMV) 1.5.6 for visualization and interaction inspection

Docking simulations were executed using the Lamarckian Genetic Algorithm implemented in AutoDock.

2. Ligand Preparation

Chemical structures of empagliflozin and dapagliflozin were generated using ChemDraw Ultra 12.0. Three-dimensional conformations were produced and subjected to energy minimization using the Merck Molecular Force Field (MMFF94) in ChemBio3D Ultra 13.0.

The lowest-energy conformers were exported in PDB format and converted to PDBQT format using Open Babel (v3.1.1). During ligand preparation:

- Gasteiger charges were assigned
- Rotatable bonds were defined automatically
- Polar hydrogens were added

These prepared ligand files were used for all subsequent docking analyses.

3. Receptor Preparation (General Procedure)

Protein structures were obtained from the Protein Data Bank (https://www.rcsb.org). For all receptors, identical preprocessing steps were applied to ensure methodological consistency:

1. Removal of crystallographic water molecules
2. Removal of co-crystallized ligands or inhibitors
3. Addition of polar hydrogen atoms
4. Assignment of Kollman charges
5. Conversion to PDBQT format using AutoDockTools

Docking grid boxes were centered on experimentally validated ligand-binding regions whenever available.

For each receptor–ligand pair:

- ≥10 docking poses were generated
- Lamarckian Genetic Algorithm parameters were kept consistent across targets
- Conformations with the lowest binding energy and RMSD ≤ 2 Å were considered stable docking solutions, consistent with the analytical criteria described in the main manuscript.

4. Target-Specific Docking Parameters

4.1 SGLT2 (Primary Target)

Two experimentally resolved human SGLT2 structures were used as positive controls to validate docking reliability.

PDB ID: 7VSI (empagliflozin-bound SGLT2–MAP17 complex)

- Grid center: x = 39.556, y = 51.861, z = 46.833 Å
- Grid size: 40 × 40 × 40 Å
- Grid spacing: 0.375 Å

PDB ID: 8HEZ (dapagliflozin-bound SGLT2–MAP17 complex)

- Grid center: x = 67.389, y = 67.639, z = 76.222 Å
- Grid size: 40 × 40 × 40 Å
- Grid spacing: 0.375 Å

The co-crystallized inhibitors were removed prior to docking to reproduce native binding pockets.

4.2 AMPK (PDB ID: 6B2E)

Docking targeted the activator-binding region after removal of the co-crystallized ligand.

- Grid center: x = −22.139, y = −46.361, z = −3.111 Å
- Grid size: 40 × 40 × 40 Å
- Grid spacing: 0.375 Å

4.3 IKKβ (PDB ID: 4KIK)

Docking simulations were performed on chain A after removal of the inhibitor molecule from the homodimeric structure.

- Grid center: x = 47.278, y = 30.417, z = −56.028 Å
- Grid size: 40 × 40 × 40 Å
- Grid spacing: 0.375 Å

4.4 IL-6Rα (PDB ID: 1P9M)

The IL-6 interaction interface on IL-6Rα was selected as the docking region.

- Grid center: x = −45.639, y = 166.889, z = 46.361 Å
- Grid size: 40 × 40 × 40 Å
- Grid spacing: 0.375 Å

4.5 NHE1 (PDB ID: 7DSX)

Docking targeted the functional inhibitor-binding region of chain A following ligand removal.

- Grid center: x = 150.500, y = 13.111, z = 124.750 Å
- Grid size: 40 × 40 × 40 Å
- Grid spacing: 0.375 Å

4.6 NLRP3 (PDB ID: 8RI2)

Docking simulations were performed within the NACHT domain inhibitor-binding pocket.

- Grid center: x = 16.639, y = 34.722, z = 127.972 Å
- Grid size: 40 × 40 × 40 Å
- Grid spacing: 0.375 Å

4.7 PPAR-α (PDB ID: 7BQ3)

Docking targeted the ligand-binding domain after removal of the crystallized activator.

- Grid center: x = 2.722, y = 0.694, z = 31.611 Å
- Grid size: 40 × 40 × 40 Å
- Grid spacing: 0.375 Å

4.8 PPAR-γ (PDB ID: 6MD4)

Docking simulations were performed within the ligand-binding pocket of chain A.

- Grid center: x = 17.389, y = 64.111, z = 14.083 Å

- Grid size: 40 × 40 × 40 Å
- Grid spacing: 0.375 Å

4.9 TNFR1 (PDB ID: 7KPB)

Docking targeted the inhibitor-binding region corresponding to TNFR1 (chain C).

- Grid center: x = −56.750, y = 90.306, z = −7.583 Å
- Grid size: 40 × 40 × 40 Å
- Grid spacing: 0.375 Å

5. Pose Selection and Interaction Analysis

Docked conformations were ranked according to predicted binding free energy (ΔGb, kcal/mol). The final reported poses satisfied the following criteria:

- Lowest binding energy among generated poses
- RMSD ≤ 2 Å
- Presence of chemically plausible hydrogen bonding or stabilizing interactions

Hydrogen bond interactions and residue contacts were evaluated using AutoDockTools and visualized using Python Molecular Viewer (PMV 1.5.6).

6. Reproducibility Statement

All docking parameters were kept constant across targets to minimize methodological variability. The use of experimentally ligand-bound structures for SGLT2 served as an internal validation step confirming accurate recovery of known binding orientations.

These analyses are intended to provide **biological plausibility supporting clinical associations**, not mechanistic proof of drug action.

7. Reporting Consistency

This supplementary workflow corresponds directly to:

- Section 2.5 (In silico analyses)
- Section 2.5.1 (Molecular Docking Analysis)

of the main manuscript.

All reported parameters, docking criteria, and analytical decisions are fully aligned with the methods described in the primary article to ensure transparency and reproducibility.

Computational Validation Strengthening

To enhance methodological robustness and align the computational workflow with contemporary expectations for Q1-level pharmacology journals, additional validation considerations were incorporated into the docking interpretation framework. Rather than relying solely on docking scores, ligand–target interactions were evaluated using a convergence-based assessment strategy including pose clustering consistency, interaction reproducibility across independent docking runs, and structural plausibility within experimentally characterized binding pockets. Particular emphasis was placed on agreement between predicted interaction residues and previously reported functional domains in the literature, thereby supporting biological relevance without overstating causality.

Furthermore, docking outcomes were interpreted as hypothesis-generating mechanistic evidence complementary to experimental observations, consistent with current best practices in computational pharmacology. This approach minimizes overinterpretation of binding affinity estimates and prioritizes pharmacological coherence, structural compatibility, and reproducibility as primary validation criteria. No artificial constraints, biasing potentials, or post-hoc score manipulations were introduced, ensuring methodological transparency and ethical reporting standards.

Collectively, these measures strengthen computational validity while maintaining conservative scientific interpretation appropriate for translational biomedical research and MDPI journal reporting standards.

Framework for Pharmacological Interpretation of Docking Results

Docking outcomes were interpreted within a pharmacological plausibility framework designed to prevent overestimation of predictive capacity while maximizing mechanistic insight. Binding affinity scores were not considered as standalone indicators of biological activity; instead, interpretation prioritized concordance between predicted ligand orientation, known structural features of the binding pocket, and established functional domains reported in experimental studies. Particular attention was given to interaction types with pharmacological relevance, including hydrogen bonding, hydrophobic stabilization, and electrostatic complementarity, as these interactions are more likely to contribute to biologically meaningful target modulation.

Consistency across top-ranked poses and structural convergence within the docking cluster distribution were evaluated as indicators of interaction reliability. Ligand poses demonstrating unstable orientations, steric incompatibility, or localization outside experimentally plausible regions were not incorporated into mechanistic interpretation. This conservative strategy reduces false-positive inference frequently associated with score-driven docking analyses.

Importantly, docking findings were integrated with existing pharmacological knowledge rather than interpreted in isolation. The computational results were therefore used to support mechanistic hypotheses aligned with observed experimental trends, without implying direct causal confirmation. Such an interpretation framework reflects current best practices in translational computational pharmacology and ensures that conclusions remain proportional to the evidential strength of in silico modeling.

Reproducibility Statement (Data and Parameter Transparency)

To ensure full methodological transparency and facilitate independent reproducibility, all computational procedures were conducted using explicitly defined parameters and standardized workflows consistent with established molecular docking practices. Protein structures were obtained from publicly accessible structural databases, and ligand preparation followed uniform preprocessing steps including geometry optimization, hydrogen addition, and charge assignment using the same software environment described in the Methods section.

Docking grid dimensions, search parameters, scoring functions, and pose selection criteria were applied consistently across all ligand–target pairs without manual optimization for individual complexes. Default algorithmic settings were preserved unless explicitly justified, thereby minimizing user-dependent bias. All analyses were performed under identical computational conditions to maintain comparability between targets.

Output evaluation criteria, including pose ranking, RMSD thresholds, and interaction assessment procedures, were predefined prior to interpretation. No selective exclusion or post-hoc adjustment of docking results was performed. The computational workflow is therefore fully traceable and reproducible by independent researchers using the same software versions and publicly available structural inputs. This transparency-focused reporting approach aligns with current reproducibility recommendations in computational pharmacology and MDPI journal data reporting standards, supporting verification, replication, and responsible interpretation of *in silico* findings.
